# Case-control and genomic epidemiology characterization of SARS-CoV-2 breakthrough infections during the Delta-to-Omicron transition

**DOI:** 10.1128/mbio.02432-25

**Published:** 2026-01-08

**Authors:** Erin Yuan, Chelsea L. Hansen, Sana Tamim, Samia Kanwar, David J. Spiro, Refugio Gonzalez-Losa, Laura Conde-Ferraez, Pilar Granja-Pérez, Salha Villanueva-Jorge, Irma López-Martínez, Gisela Barrea-Badillo, André Corvelo, Samantha Fennessey, Michael C. Zody, Guadalupe Ayora-Talavera, Nidia S. Trovao

**Affiliations:** 1 Harvard University 1812, Cambridge, Massachusetts, USA; 2 Division of International Epidemiology and Population Studies, Fogarty International Center, National Institutes of Health 2511, Bethesda, Maryland, USA; 3 PandemiX Center of Excellence, Roskilde University 6976, Roskilde, Denmark; 4 National Institute of Health, Islamabad, Pakistan; 5 Department of Paediatrics and Child Health, The Aga Khan University 9615, Karachi, Pakistan; 6 Laboratorio de Virología, CIR-Biomédicas, Universidad Autónoma Yucatán, Mérida, México; 7 Laboratorio Estatal de Salud Pública, Servicios de Salud de Yucatán, Mérida, México; 8 Instituto de Diagnóstico y Referencia Epidemiológicos, Dirección General de Epidemiología 61694, Mexico City, Mexico; 9 New York Genome Center 377591, New York, New York, USA; Johns Hopkins University, Bloomberg School of Public Health, Baltimore, Maryland, USA

**Keywords:** phylodynamics, epidemiology, COVID-19, surveillance

## Abstract

Severe acute respiratory syndrome coronavirus 2 (SARS-CoV-2) vaccines reduced severe coronavirus disease 2019, but variants like Delta and Omicron caused widespread breakthrough infections (BIs). Mexico, offering diverse vaccines, and its Yucatán region, a major travel hub, provide a unique setting to study BIs. We characterized SARS-CoV-2 BIs in Yucatán during the Delta-to-Omicron transition (September 2021–January 2022), assessing disease severity, symptoms, and viral transmission dynamics using epidemiological and genomic data. A case-control study using health system data (*n* = 13,325) compared outcomes in BIs (*n* = 5,183) versus unvaccinated infections (UIs; *n* = 8,142) via logistic regression, also comparing Delta versus Omicron waves. Phylodynamic modeling of 205 BI sequences, contextualized globally (*n* = 1,152 total), reconstructed the evolutionary history and transmission routes. Vaccination significantly reduced hospitalization (odds ratio [OR] = 0.38) and death (OR = 0.45) in BIs compared to UIs. Omicron infections were less severe than Delta (hospitalization OR = 0.60; death OR = 0.33) and presented with less loss of smell/taste but more upper respiratory symptoms. Phylodynamics revealed numerous introductions (17 Delta, 36 Omicron). Delta BIs in Yucatán originated mainly from within Mexico, Guatemala, Europe, and the United States. Omicron BIs in Yucatán had more diverse origins including from North and South America and Africa, coinciding with eased travel restrictions. Vaccines maintained protection against severe outcomes during the Delta and Omicron waves in Yucatán. Omicron, though less severe, showed enhanced transmissibility with increased introductions linked to relaxed public health measures. Findings highlight the critical role of continued vaccination, genomic surveillance, adaptive policies, and cross-border collaboration for pandemic preparedness and health security.

**IMPORTANCE:**

Our understanding of severe acute respiratory syndrome coronavirus 2 breakthrough infections in Latin America is limited, specifically in regions with unique epidemiological dynamics. In this study, we fill a knowledge gap by characterizing these infections in Yucatán, Mexico, a major international travel hub with one of the world's most diverse vaccine rollouts, during the critical transition from the Delta variant to the Omicron variant. The translational importance of our investigation is twofold. First, through case-control data analysis, we provide robust, real-world evidence that vaccination significantly reduced the risk of hospitalization and death, offering crucial data to support ongoing vaccination campaigns against emerging variants. Second, by combining epidemiological data with phylodynamic analysis, we demonstrate a direct link between the easing of public health restrictions and the increased number and diversity of viral introductions that sparked the Omicron wave. This highlights the critical importance of coordinating genomic tracking with public health policy to mitigate the spread of future pandemic threats and strengthen global health security.

## INTRODUCTION

Vaccines against severe acute respiratory syndrome coronavirus 2 (SARS-CoV-2) have been vital in reducing severe outcomes of the coronavirus disease 2019 (COVID-19), such as hospitalizations and death. Widely used SARS-CoV-2 vaccines during the pandemic include those manufactured by AstraZeneca, CanSino, Gamaleya, Janssen, Moderna, Novavax, Pfizer, Sinopharma, and Sinovac. Of these, mRNA vaccines have generally reported the highest vaccine effectiveness (1). However, vaccines are not effective at preventing infection against all variants of SARS-CoV-2, particularly the Delta and Omicron variants of concern (VOCs) (2, 3).

The Delta and Omicron variants of SARS-CoV-2 have distinct characteristics that have significantly impacted the global response to the COVID-19 pandemic. The Delta variant is 40%–60% more contagious than the Alpha variant, largely due to mutations such as E484Q, L452R, and P681R, which enhance its infectivity (4), while the Omicron is associated with milder symptoms and a 15%–80% reduced risk of hospitalization compared to Delta, with most cases being asymptomatic or mild. Though vaccination was effective against the Delta variant, some had the ability to evade immune responses, necessitating booster doses to maintain protection (4). Despite vaccination, Omicron led to a higher rate of breakthrough infections (BIs), although these cases are generally less severe (5, 6). These differences have influenced public health strategies and vaccine development efforts worldwide.

These VOCs have posed a global public health challenge in managing the COVID-19 pandemic due to re-infections (RIs) and BIs (7, 8). RIs are defined as the reappearance of clinical symptoms of COVID-19 with a confirmed molecular test for SARS-CoV-2 within 90 days of exposure or the genetic identification by sequencing of a different virus from the previous infection (9, 10). BIs are defined as the presence of viral antigen or viral RNA in the clinical sample of an individual more than 14 days after vaccination (11). RIs and BIs have occurred during the entire span of the SARS-CoV-2 evolutionary journey from wildtype to Omicron and its subvariants (12–14).

BIs were reported across the world even in populations that received multiple vaccinations, including India, the EU, and the United States (15–18). The upward surge of RIs and BIs was believed to be due to the uneven distribution of vaccination across different populations (19, 20). Even with its leading vaccination rate and a pre-Delta vaccine effectiveness of 94.3%, Israel saw significant breakthrough and reinfections during the Delta surge, as vaccine effectiveness plummeted to 30%–64% (21). Multiple confounding factors, such as host biological factors, improper administration or storage of vaccines, viral evolution, and variable vaccine immunogenicity, are likely to have played a significant role in BIs (22, 23). Belgium and France reported BIs and RIs due to Omicron VOC subvariant BA.1 and BA.2 (18, 24). BI or RI risk with Omicron has been reported to be sixfold higher than that with other SARS-CoV-2 variants (25). However, it has been suggested that increased BIs or RIs due to Omicron generally were associated with milder infections than previous variants (17).

Latin America was not the exception to report BIs during the COVID-19 pandemic. BIs were reported from Colombia or Brazil with a P1 or Gamma variant (26–28). Countries like Peru, Argentina, Chile, and Brazil also reported BIs with the Omicron surge (29–31).

Mexico is an important area for analysis of the COVID-19 pandemic because its governmental health authority, the Federal Commission for Protection against Health Risks, offered one of the most diverse selections of vaccines in the world (32). Yet, there remains a lack of literature on vaccination and the impact of COVID-19 on the Mexican population. The scope of this work centers on Mexico’s southeastern Yucatán peninsula because the region continued to experience a significant influx of tourists and remained highly connected to the United States and the rest of the world throughout the pandemic (33). Thus, Yucatán had complex viral dynamics in terms of the import of new variants and the contribution to the subsequent spread of the variants throughout Mexico (34).

Mexico declared a state of emergency on 30 March 2020 and instituted a national-level lockdown until 31 May 2020, which included closure of schools and businesses. The federal lockdown was lifted on June 1st and implemented an epidemiological traffic light risk based on colors by the levels of COVID-19 reporting. However, local non-pharmaceutical interventions were implemented by all 32 Mexican state governments and municipalities based on guidance from local public health committees. Restrictions gradually lifted through Fall 2020 and Spring 2021 (35). In Yucatán, no further restrictions were put in place in response to the rise in cases and deaths resulting from the introduction of the Delta or Omicron variants; however, the use of face masks was compulsory in all public spaces open and closed, hospitals, public transport, and schools until 12 May 2022 (36).

Vaccination in Mexico, particularly in Yucatán, began at-scale deployment in April 2021. Vaccine deployment was implemented by age group and risk, with health personnel first, followed by individuals older than 60 years, and finally individuals older than 18 years. In December 2021, vaccine coverage reached 84% of Mexicans older than 5 years with at least one dose (37). Nevertheless, cases of BIs were reported from hospitalized patients and the general population (38, 39). At the population level, the case fatality rate of Mexican frontline healthcare workers due to COVID-19 was the highest among low- and middle-income countries (40, 41).

Here, we present a case-control study of BIs in healthcare workers and the general public from various public and private sector healthcare institutes. We assess (i) disease severity and symptom profiles in vaccinated versus unvaccinated individuals with SARS-CoV-2 infection; (ii) differences in disease severity and symptom profiles between the Delta and Omicron BA.1 VOCs; and (iii) the evolutionary history and transmission dynamics of SARS-CoV-2 VOC BIs in Yucatán, Mexico, by combining epidemiological and genomic data within a phylodynamic framework.

Understanding the transmission dynamics of respiratory viruses in Mexico through genomic epidemiology is also important for U.S. health security, as the close geographic proximity and high volume of travel and trade between the two countries create a shared public health landscape that facilitates pathogen spread. This research provides insights into the effectiveness of vaccination, the impact of emerging variants, and the role of international mobility in shaping pandemic response strategies.

## RESULTS

### Case-control analysis

Analysis included data from 13,325 individuals (BIs: *n* = 5,183 with 92% Delta and 8% Omicron; unvaccinated infections [UIs]: *n* = 8,142 with 93% Delta and 7% Omicron; Table 1). The majority of participants were <40 years of age (54%), resided in Merida (89%), worked in an office setting (64%), and reported no underlying health conditions (77%). Of the 5,183 participants who reported vaccination, most (65%) had received viral vector vaccines (AstraZeneca, CanSino, Gamaleya, and Janssen), and 71% had completed their primary series. Fewer than 12% required inpatient hospitalization or died.

**TABLE 1:** Demographic characteristics of study participants and sequences^*a*^^,^^*b*^

Characteristics	Included enrollments, *n* = 13,325				Total sequenced, *n* = 205		
	*n*	All, *n* = 13,325	BI cases, *n* = 5,183	UI cases, *n* = 8,142	*n*	SINAVE, *n* = 64	UADY, *n* = 141
Variants	13,325				205		
Delta		12,307 (92%)	4,752 (92%)	7,555 (93%)		64 (100%)	27 (19%)
Omicron		1,018 (8%)	431 (8%)	587 (7%)		0 (0%)	114 (81%)
Age groups	13,325				200		
<18		0 (0%)	0 (0%)	0 (0%)		0 (0%)	5 (3.7%)
18–29		3,789 (28%)	1,269 (24%)	2,520 (31%)		15 (23%)	14 (10%)
30–39		3,401 (26%)	1,388 (27%)	2,013 (25%)		12 (19%)	18 (13%)
40–49		2,569 (19%)	1,063 (21%)	1,506 (18%)		19 (30%)	46 (34%)
50–59		1,547 (12%)	599 (12%)	948 (12%)		9 (14%)	35 (26%)
≥60		2,019 (15%)	864 (17%)	1,155 (14%)		9 (14%)	18 (13%)
Sex	13,325				205		
Female		6,572 (49%)	2,648 (51%)	3,924 (48%)		35 (55%)	75 (53%)
Male		6,753 (51%)	2,535 (49%)	4,218 (52%)		29 (45%)	66 (47%)
Location	13,325				205		
Merida		11,916 (89%)	4,394 (85%)	7,522 (92%)		44 (69%)	141 (100%)
Tizimin		763 (5.7%)	431 (8.3%)	332 (4.1%)		14 (22%)	0 (0%)
Ticul		646 (4.8%)	358 (6.9%)	288 (3.5%)		6 (9.4%)	0 (0%)
Job	13,325				205		
HCW		900 (6.8%)	696 (13%)	204 (2.5%)		31 (48%)	0 (0%)
Home		2,356 (18%)	965 (19%)	1,391 (17%)		17 (27%)	0 (0%)
Office		8,537 (64%)	2,760 (53%)	5,777 (71%)		5 (7.8%)	0 (0%)
Other		476 (3.6%)	239 (4.6%)	237 (2.9%)		4 (6.3%)	0 (0%)
School		577 (4.3%)	308 (5.9%)	269 (3.3%)		1 (1.6%)	141 (100%)
Tradesmen		479 (3.6%)	215 (4.1%)	264 (3.2%)		6 (9.4%)	0 (0%)
Indigenous	13,325				64		
0		12,930 (97%)	4,925 (95%)	8,005 (98%)		55 (86%)	0 (NA%)
1		395 (3.0%)	258 (5.0%)	137 (1.7%)		9 (14%)	0 (NA%)
Conditions	13,325				64		
0		10,218 (77%)	3,578 (69%)	6,640 (82%)		26 (41%)	0 (NA%)
1		3,107 (23%)	1,605 (31%)	1,502 (18%)		38 (59%)	0 (NA%)
Vaccine	13,325				64		
Complete		3,659 (27%)	3,659 (71%)	0 (0%)		53 (83%)	0 (NA%)
Incomplete		1,524 (11%)	1,524 (29%)	0 (0%)		11 (17%)	0 (NA%)
None		8,142 (61%)	0 (0%)	8,142 (100%)		0 (0%)	0 (NA%)
Vaccine name	13,260				205		
None		8,142 (61%)	0 (0%)	8,142 (100%)		0 (0%)	0 (0%)
mRNA		1,370 (10%)	1,370 (27%)	0 (0%)		39 (61%)	23 (16%)
Viral vector		3,352 (25%)	3,352 (65%)	0 (0%)		22 (34%)	117 (83%)
Whole or protein		396 (3.0%)	396 (7.7%)	0 (0%)		3 (4.7%)	1 (0.7%)
Vaccine time	5,183	91 (47, 153)	91 (47, 153)	NA	64	154 (92, 218)	NA
Outcome	13,325				205		
Outpatient		11,820 (89%)	4,576 (88%)	7,244 (89%)		31 (48%)	141 (100%)
Inpatient		651 (4.9%)	259 (5.0%)	392 (4.8%)		14 (22%)	0 (0%)
Death		854 (6.4%)	348 (6.7%)	506 (6.2%)		19 (30%)	0 (0%)
num_syms^*c*^	13,325	5.00 (4.00, 7.00)	5.00 (4.00, 7.00)	5.00 (4.00, 6.00)	205	9.00 (6.00, 10.00)	6.00 (4.00, 7.00)

^*a*^ The table presents the demographic characteristics of the study participants, including both BIs and UIs, as well as the subset of cases with sequenced viral genomes from the SINAVE and UADY databases.

^*b*^ BI cases: breakthrough infections; UI cases: unvaccinated infections; *n*: number of participants; NA: not available; HCW: healthcare worker; SINAVE: National Epidemiological Surveillance System database; UADY: Autonomous University of Yucatán.

^*c*^ num_syms; number of symptoms.

**Fig 1:**
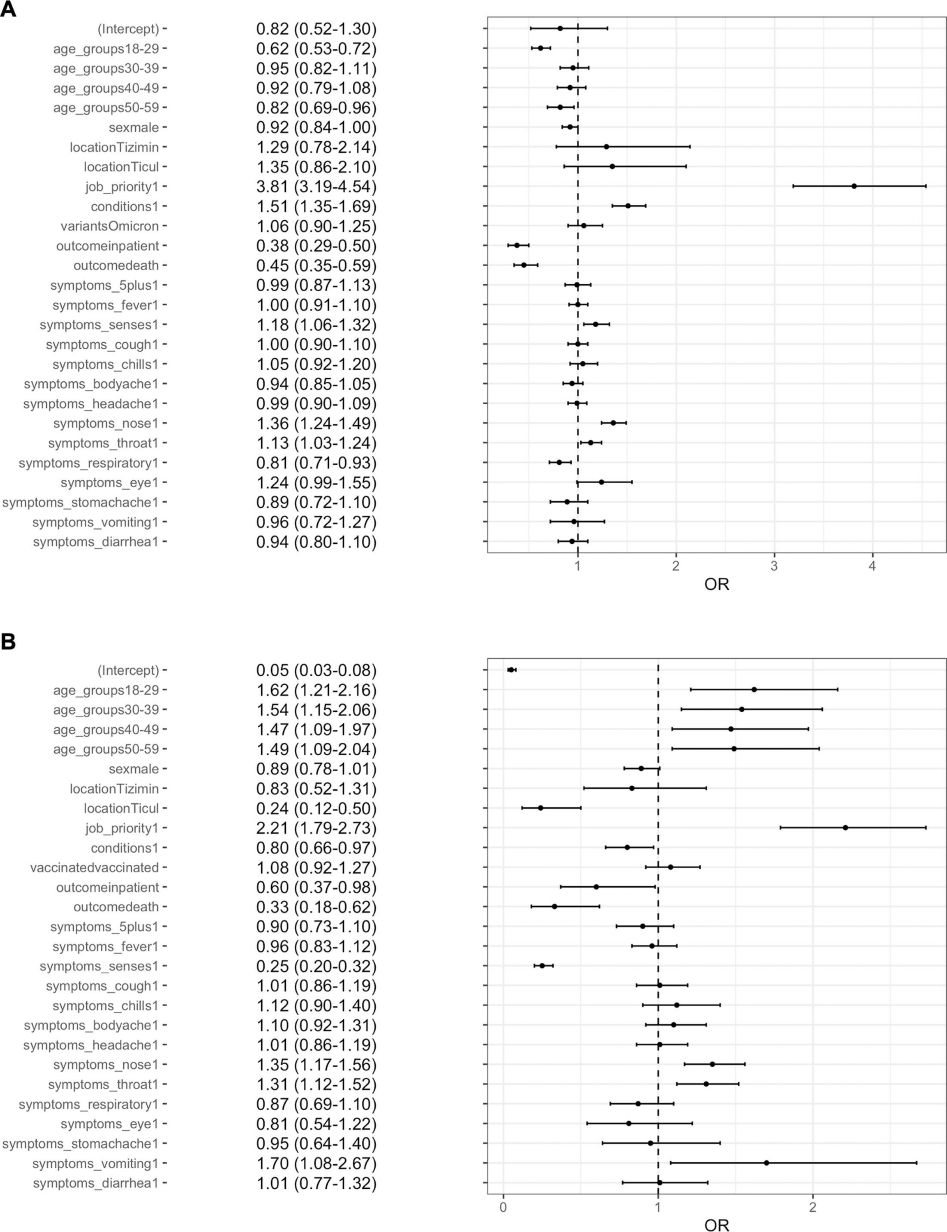
(**A**) Mixed-effects multiple logistic regression was performed to understand how vaccination affects disease severity and symptom profiles among individuals who tested positive for a SARS-CoV-2 infection while controlling for age, sex, location, occupation, prior conditions, and VOC. ORs are shown for both variables likely to influence vaccination status and outcomes, followed by the range of the 95% CI, where ORs above 1 indicate increased associations, while ORs below 1 indicate decreased associations. (**B**) Logistic regression was performed to understand how the type of VOC (Delta vs Omicron) affects disease severity and symptom profiles among cases while controlling for age, sex, location, occupation, prior conditions, and vaccination status. ORs are again shown for both control and outcome variables.

To understand the impact of vaccination on disease presentation and severity, we compared BIs to UIs while controlling for variables likely to impact vaccination status. We found that cases who were working in a high-priority occupation (healthcare workers and teachers) were 3.81 (95% confidence interval [CI] 3.19–4.54) times more likely to be vaccinated than cases in other occupations (Fig. 1A). Individuals with high-risk conditions were also more likely to be vaccinated (odds ratio [OR] = 1.51, 95% CI 1.35–1.69). Younger individuals were less likely to be vaccinated than individuals ≥60 years, and this was most pronounced in individuals 18–29 years, the youngest age group included (OR = 0.62, 95% CI 0.53–0.72). After controlling for these variables, we did not find significant variation in symptom presentation between vaccinated and unvaccinated SARS-CoV-2 infections. BI cases were slightly more likely to report loss of smell and taste, runny nose, and sore throat (ORs between 1.1 and 1.4), and less likely to report more severe symptoms such as difficulty breathing (OR = 0.81, 95% CI 0.71–0.93) than unvaccinated cases. However, differences were more pronounced for severe outcomes. BI cases were much less likely to require hospitalization (OR = 0.38, 95% CI 0.29–0.50) or have a fatal outcome while hospitalized (OR = 0.45, 95% CI 0.35–0.59) than unvaccinated cases. Results from sensitivity analysis limiting BI cases to those who had completed their primary vaccination series were broadly consistent with these findings (Fig. S2).

When comparing Omicron to Delta cases, Omicron cases were less likely to report loss of smell and taste (OR = 0.25, 95% CI 0.20–0.32), require hospitalization (OR = 0.60, 95% CI 0.37–0.98), or experience a fatal outcome while hospitalized (OR = 0.33, 95% CI 0.18–0.62). Omicron cases were more likely to be younger (OR = 1.62, 95% CI 1.21–2.16) and report upper respiratory symptoms including runny nose and sore throat (OR = 1.35, 95% CI 1.17–1.56) (Fig. 1B).

### Genomic epidemiology

#### Transmission dynamics

**Fig 2:**
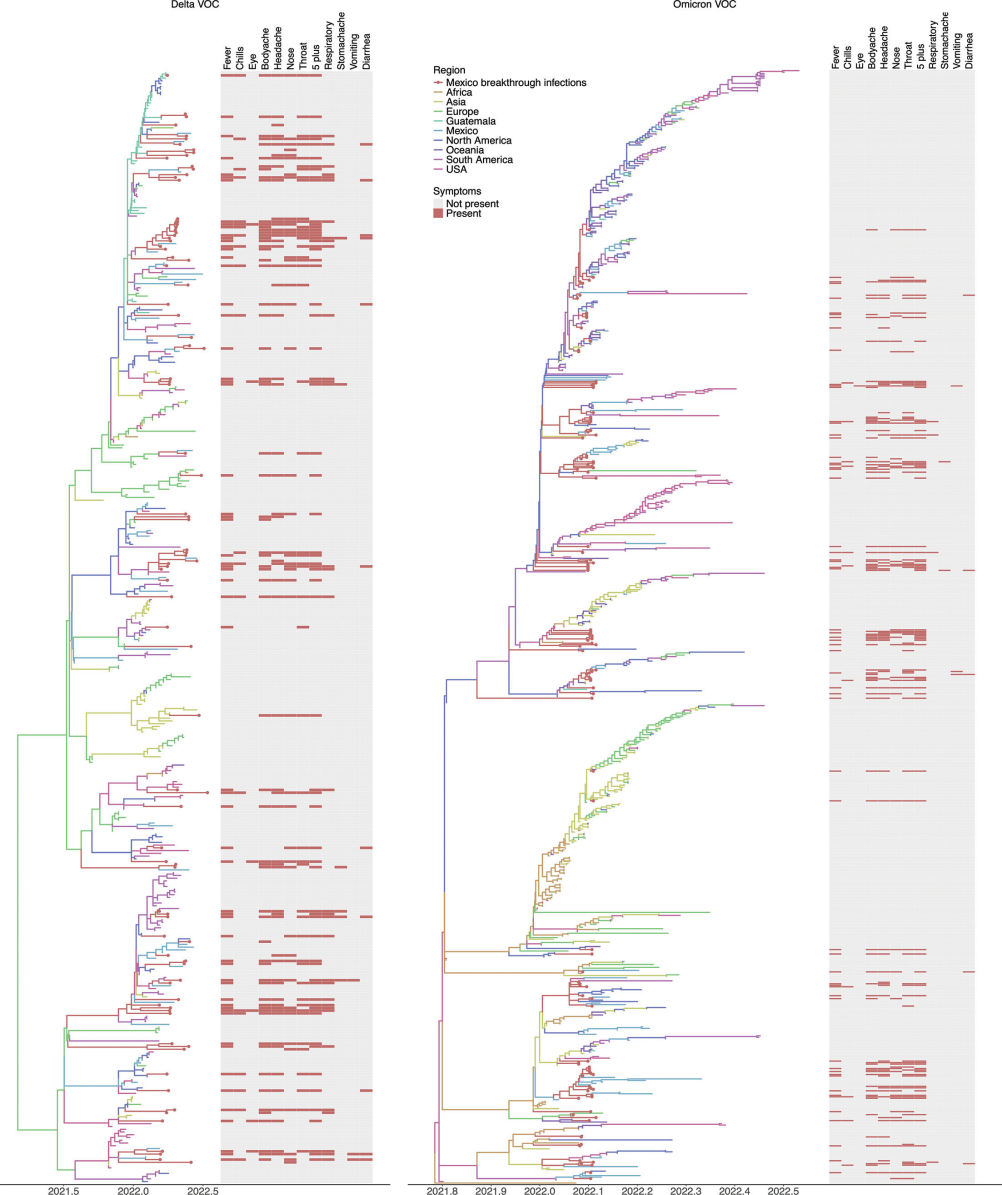
Evolutionary and spatio-temporal history of SARS-CoV-2 BIs in Yucatán, Mexico. Time-scaled phylogenetic tree of Delta VOC (left) and Omicron VOC (right). The branches are colored according to the reconstructed location at the ancestral nodes. Sequences of BIs in Yucatán are depicted as red circles. Heatmaps depict the presence (red) or absence (gray) of symptoms.

We investigated the relationship between SARS-CoV-2 evolution, spatial-temporal transmission patterns, and clinical presentation. We reconstructed a phylogeographic tree based on whole-genome sequences from infected individuals (Fig. 2). A heatmap was then generated to visualize the presence or absence of key symptoms for each study sequence. Both Delta and Omicron trees reveal several distinct clades of SARS-CoV-2 circulating in the study population, indicating multiple introductions into Mexico, particularly leading to BIs. As described above, the Omicron clades appear to have a stronger association with milder symptoms, fewer occurrences of loss of smell and taste, and more upper respiratory symptoms compared to those belonging to the Delta variant, where more lower respiratory tract infections were observed. A strong correlation among symptoms also exists since patients who experienced one symptom appear likely to have experienced many regardless of the clade of virus.

**Fig 3:**
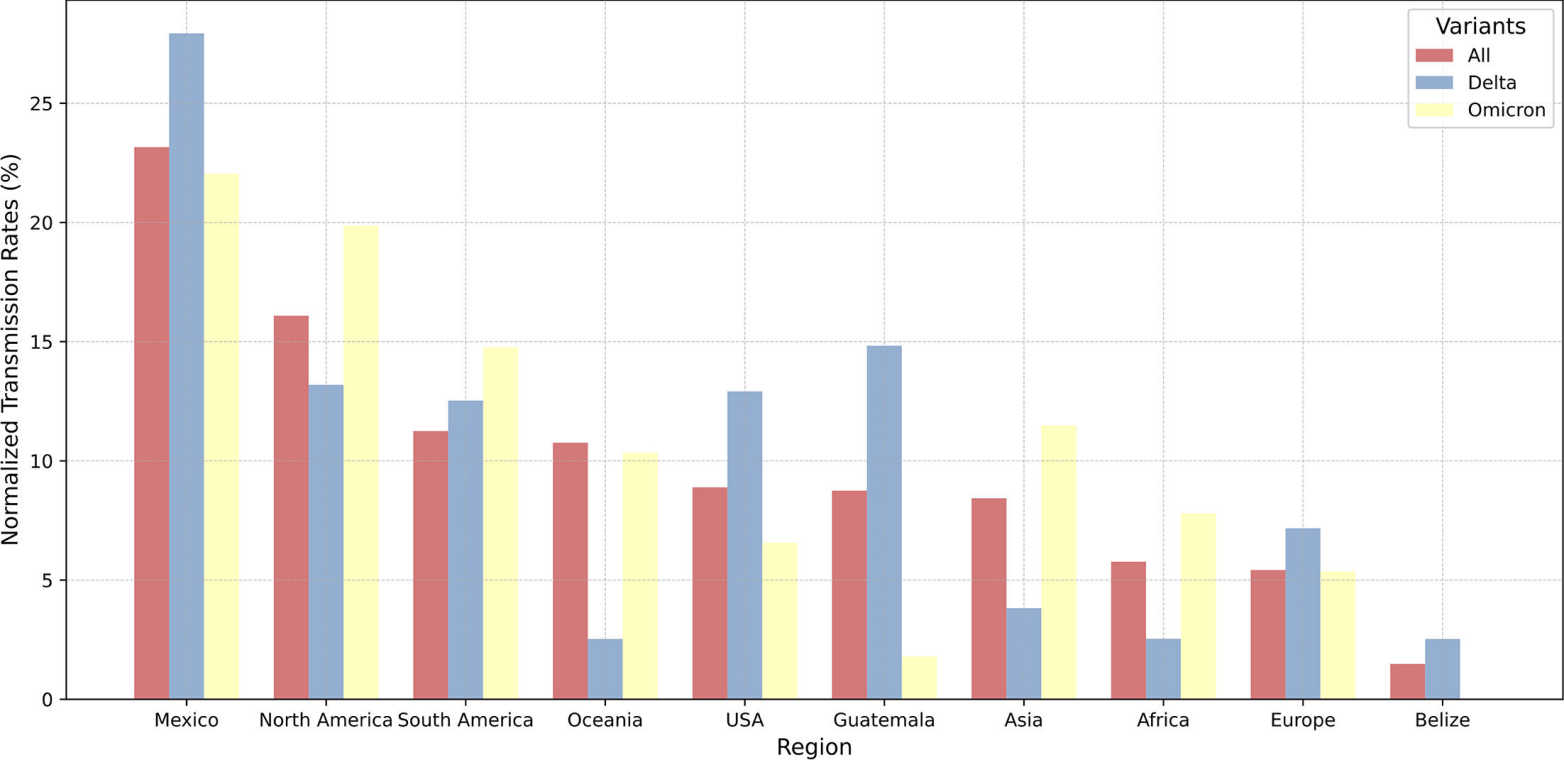
Source of SARS-CoV-2 VOCs leading to BIs in Yucatán, Mexico. Normalized transmission rates (as percentage) across regions estimated with the “mugration” model (see Table S1).

Phylogeographic reconstruction of all lineages that caused BIs in Yucatán (Fig. 2 and 3) revealed that close to a quarter of all infections (23%) were acquired locally from locations across Mexico (Fig. 3, All; Table S1, All). Oceania (11%), South America (11%), and other North American countries (16%) contributed close to 38% of all viruses, leading to BIs in Yucatán. These viruses were identified as Delta VOC and Omicron VOC. We sought to assess the contribution of various locations in generating BIs of different lineages and across various phases of the pandemic in Yucatán. For BIs caused by the Delta VOC, our reconstructions estimated that 28% were acquired from locally circulating SARS-CoV-2 in Mexico and 15% had origins in Guatemala (Fig. 3, Delta; Table S1, Delta). BIs caused by the Omicron VOC were seeded by viruses circulating locally (22%), North America (20%), and South America (15%; Fig. 3, Omicron; Table S1, Omicron).

**Fig 4:**
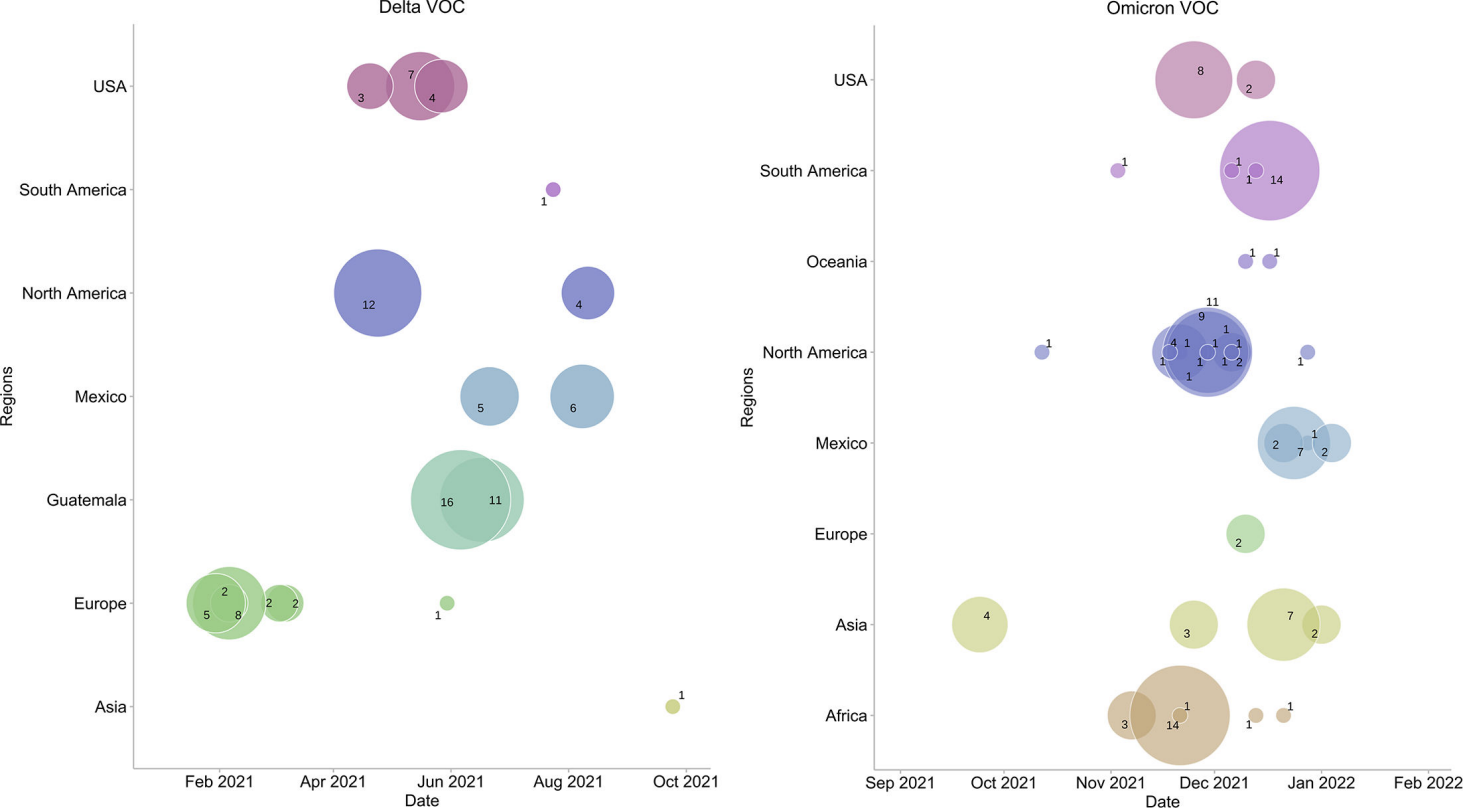
Timeline of introductions of VOC/VOI that led to BIs in Mexico. Bubbles represent lineage-specific viral introductions (left, Delta VOC; right, Omicron VOC) over time of the most recent common ancestor inferred from BI-specific clades in the phylogenetic tree. The circle size depicts the number of sequenced BIs that resulted from each introduction.

The transmission of SARS-CoV-2 into Yucatan, Mexico, was characterized by at least 17 introductions of the Delta VOC and 36 introductions of the Omicron VOC during the study period. The most significant period of viral introductions leading to BIs caused by the Delta VOC in Mexico occurred between May 2021 and July 2021, as depicted in Fig. 4, left. Among the identified introductions, two from Guatemala and three introductions from the United States contributed significantly to the largest outbreaks. These introductions resulted in the detection of 27 cases with origins in Guatemala and 14 cases with origins in the United States. The origins of the Delta variant BIS cases were diverse: 35.2% tracing back to Europe; 17.6% tracing to the United States, 11.76% tracing to Guatemala, Mexico, and North America each; and 5.8% tracing to South America and Asia each. In contrast, and likely worsened by the relaxation of travel restrictions, we estimated that the majority of viral introductions causing Omicron BIs, during the period from November 2021 to January 2022, originated from North America (38.88%), followed by Africa (13.88%), Mexico, South America, and Asia (11.11% each), Oceania and the United States (5.52%), and Europe (2.71%) (Fig. 4, right).

## DISCUSSION

The Yucatán region of Mexico is a major tourist destination and is therefore globally connected. In this study, we characterized the epidemiology, evolution, and transmission dynamics of SARS-CoV-2 BIs in Yucatán during the Delta and Omicron waves to shed light on the interplay between the viral evolution and control measures and to inform future public health guidelines, including the coordination of genomic surveillance efforts in Mexico and neighboring nations, as the COVID-19 pandemic continues to evolve.

Consistent with the vaccination strategy in Yucatán, we found that those working in high-priority occupations and living with prior conditions were more likely to be vaccinated. For vaccinated individuals who became infected, we observed a reduction in severe outcomes compared to unvaccinated individuals, as demonstrated by a 62% lower odds of requiring hospitalization or a 55% lower odds of mortality as an inpatient. Further stratifying this by the SARS-CoV-2 variant responsible for the infection, we found that Omicron VOC cases had 40% lower odds of requiring hospitalization and 67% lower odds of a fatal outcome than Delta VOC cases after adjusting for vaccination status. Our estimates align with the patterns of significantly reduced risk of severe disease for Omicron VOC (42). Finally, we noted a shift in symptoms from the Delta-to-Omicron wave, as Omicron VOC cases were less likely to report loss of smell and taste and instead more frequently reported upper respiratory symptoms of runny nose and sore throat than Delta cases. Our results are in line with previous studies that found loss of smell more prevalent during the Delta VOC wave (43), sore throat symptoms during the Omicron VOC wave (44), and higher reporting of influenza-like symptoms, like runny nose, during the Omicron VOC wave (45).

We also explored the evolutionary and transmission patterns of SARS-CoV-2 in Yucatán. Geographic ancestral reconstruction revealed that different locations contributed to the genetic diversity of the Delta and Omicron viruses found in Mexico, shedding light on the intricate dynamics of the pandemic. Europe (35.2%), United States (17.6%), and Guatemala (11.7%) were identified as the primary sources of Delta VOC BI cases in Mexico. Compared to the diverse origins of Omicron VOC BI cases, Delta VOC BIs were introduced from a smaller number of locations, likely attributable to the stricter COVID-19 restrictions in place at the time.

Globally, the largest COVID-19 wave was caused by the Omicron VOC, which was brought on by a spike of imported infections. The easing of lockdown conditions and travel restrictions likely facilitated the viral introductions between November 2021 and January 2022 from several places, including Africa, North America, and South America, that led to the Omicron VOC BIs. In Mexico, the reduction of the tight, lengthy, city-wide lockdown and targeted measures put in place in 2020 enabled transmission of the Omicron VOC upon its introduction (34). The study highlights the increased transmissibility and potential for rapid dissemination of the Omicron variant compared to Delta. Several factors may contribute to this observed difference, including genetic mutations that enhance viral fitness or increased human-to-human transmission rates (46). The higher number of Omicron introductions into Yucatán (*n* = 36), compared to Delta (*n* = 17), also indicates greater genetic diversity within this variant, which continues to lead to the emergence of subvariants with diverse characteristics (47).

There is a paucity of research investigating BIs of SARS-CoV-2 within populations in Latin America, including Mexico. Data from Brazil, one of the most affected countries, report BIs in different populations with Gamma and Omicron variants (27, 28). In Peru and Argentina, BIs were reported in HCW during the Omicron surge besides receiving homologous or heterologous vaccination (29, 31), and data from Chile reported BI in the pediatric population also during the Omicron surge after CoronaVac vaccination (30). Nonetheless, in agreement with our findings, Galán-Huerta et al. (38) also investigated study populations in Mexico, where most BIs were of the Delta variant. Specifically, the B.1.617.2 lineage of Delta was most common in ambulatory (outpatient) vaccinated individuals (*P* < 0.01), while the AY.4 lineage of Delta was predominant in hospitalized patients, regardless of vaccination status (*P* = 0.04). Their phylogenetic analysis showed multiple introductions of Delta into the region, with some evidence of local transmission chains. The authors also noted that the increasing COVID-19 cases in Mexico during the study period coincided with the rise of the Delta variant. Though not focused on Mexico, other studies also demonstrate that the emergence and spread of new variants, particularly during the Omicron wave (47, 48), may play a role in the occurrence of BIs, as they are more likely to infect vaccinated individuals who may not have received booster shots that target the new variant.

While genomic surveillance in Mexico faces challenges due to a lack of infrastructure and resources, as well as a lack of collaborative effort from public health organizations (49), our study highlights how critical these efforts are in tracking new variants and infections. In particular, while other studies have analyzed genome sequences from early circulating lineages (50), and as the Omicron VOC increased in prevalence (51), our study fills a gap in the timeline during the Delta wave when genomic surveillance was limited.

Our phylodynamic reconstructions also suggest that public health measures likely led to fewer regions contributing to the transmission of the Delta VOC in Mexico. Conversely, our analysis suggests that the easing of these interventions—along with more lenient travel regulations and vaccine reluctance—favored the introduction and dissemination of the Omicron VOC in the country (51).

There are several limitations to this study. In the epidemiological modeling, our data did not include information on prior infections, which may have led to an underestimation of the effect of vaccines on symptom severity. The data set also did not permit statistical analysis of the differences between specific vaccine types, a challenge given Mexico’s diverse vaccine portfolio. However, we note that viral vector vaccines were the most common type received by our vaccinated participants (65%) and that overall vaccines still provided significant protection against severe COVID-19 in the BI group. During the Omicron wave, hybrid immunity by vaccination and prior infection provided protection against RI and COVID-19 severity in the Mexican population (39). Additionally, we categorized variants as Delta and Omicron according to the date of infection because sequencing to identify the variant was not available for every sample. Due to the rapid displacement of Delta by Omicron within a span of 2 weeks, any misclassification of samples should be minimal. The data collection timeline also only captured the beginning of the Omicron wave in Mexico, and further time points may more strongly confirm our Delta versus Omicron comparisons. Another factor missing from the data was the time since vaccination, which may influence disease severity or risk of infection due to waning immunity. However, all patient samples were taken within 100 days of receiving the most recent vaccine dose, and so this was not considered in our analyses (52, 53). Finally, the data may exhibit bias because it only captures patients who presented for care and does not capture information on patients who may have experienced mortality as a result of COVID-19 after leaving the healthcare facility (either inpatient or outpatient), thus potentially overlooking a significant segment of the broader Yucatán population in the conclusions. Furthermore, bias may be introduced since the data rely on patient self-reported symptoms, which could be influenced by their knowledge of a positive SARS-CoV-2 test result. This may lead to an over-reporting of symptoms or a perception of a stronger correlation among symptoms than what truly exists in the population.

The phylodynamics reconstructions are also limited by the small sample size as only a small subset of cases was sequenced. The small data set restricts the understanding of the real extent of viral evolution and transmission of viruses causing BIs in Mexico. The absence of symptom information for the background sequences is another drawback that would have allowed a more thorough examination of the severity of BIs and their association with SARS-CoV-2 evolutionary dynamics. We also have an uneven genome sampling with a number of viral genomes sequenced in space and time that might not always reflect the geographic distribution and extent of the viral transmission. Lastly, our genomic analysis relies on sequenced samples coming from two distinct populations with varying demographic and sampling factors: the SINAVE database (representing the broader case-control population, which was predominantly Delta) and additional samples from the UADY population (which were mostly Omicron). This discrepancy can introduce biases in our phylodynamic reconstructions, making it difficult to definitively conclude whether the observed differences in transmission patterns and geographic origins are solely due to the distinct dynamics of the two variants or are partly influenced by variations in the sampled populations' characteristics.

This work examines the epidemiology and diversity of SARS-CoV-2 BIs in Yucatán, Mexico, during the Delta and Omicron waves. This study has significant implications for health security, as it highlights the role of international travel in introducing and sustaining SARS-CoV-2 VOCs. The findings demonstrate how travel restrictions influenced the spread of the Delta variant, while the relaxation of such measures facilitated the rapid introduction and transmission of Omicron, underscoring the importance of adaptive border control policies. The effects of travel restriction have been observed across various settings (54–57). Additionally, the study reinforces the protective effect of vaccination against severe disease outcomes, providing evidence to support continued vaccination efforts and booster campaigns in the United States and elsewhere to mitigate future outbreaks. The genomic surveillance data also emphasize the need for enhanced pathogen tracking, allowing for early detection of variants that may pose a heightened risk to U.S. and Mexican public health systems. By integrating epidemiological and genomic approaches, this research offers a model for improving pandemic preparedness and response strategies in Mexico, the United States, and other nations with strong regional connectivity.

## MATERIALS AND METHODS

### Data

Data come from individuals testing positive for SARS-CoV-2 after presenting with COVID-like symptoms in the Yucatán state of Southern Mexico at one of its health system institutions. These include private clinics in the capital city of Mérida and public health institutions (IMSS, SSA, ISSSTE, and SEDENA), with data aggregated as part of the SINAVE database. Upon visiting the hospital or health center, patients reported demographic information (age, gender, sex, and ethnicity), residency, occupation, symptoms, comorbidities, medications, and vaccination history (Supplementary file). The SINAVE database consists of 46,453 records of confirmed COVID-19 cases diagnosed through a real-time RT-PCR or antigen rapid test between 1 September 2021 and 10 January 2022, during which Delta and Omicron BA.1 VOCs were the predominant variants circulating.

From this data set, individuals who tested negative (*n* = 24,953), were <18 years of age (*n* = 4,547), had an invalid SARS-CoV-2 test result or unknown vaccination status (*n* = 3,096), were pregnant (*n* = 558), or reported travel in the past 14 days (*n* = 19) were excluded from analyses (Fig. S1). BIs referred to individuals with a positive SARS-CoV-2 test result who reported receiving 1 or 2 doses of a vaccine (from any manufacturer) for more than 14 days prior to the date of their test, and UIs were defined as individuals with positive tests with no evidence of vaccination.

The data set of included participants was used to understand the epidemiological characteristics of a subset of 205 SARS-CoV-2 samples of BIs from which viral genetic sequences were generated. To maximize the size of the genomic data set, we included samples that were excluded from the case-control analysis and obtained additional sequences from the UADY employees and respective family to supplement the SINAVE database.

### Case-control analysis

To understand the impact of vaccination on disease severity and symptom profiles, we used mixed-effects multiple logistic regression to compare BIs to UIs. In this analysis, all participants had a positive SARS-CoV-2 test result, and the dependent variable was vaccination status. The fixed-effects independent variables included clinical outcome (recovered as an outpatient, recovered as an inpatient, died as an inpatient) and 14 individual symptoms (partial or full loss of taste or smell; cough; fever; chills; conjunctivitis; body aches in the form of muscle aches, joint aches, or malaise; headache; runny nose; sore throat; lower respiratory problems including difficulty breathing, rapid respiration, chest pain, or skin discoloration; stomachache; vomiting; diarrhea). We also included fixed effects for variables likely to influence vaccination status (File S1). These included age group in years based on vaccine eligibility (18–29, 30–39, 40–49, 50–59, and ≥60), sex (female, male), location of patient’s residence within the three jurisdictions of the Yucatán State Health Service (with main localities in Mérida, Tizimin, or Ticul), priority occupational status (healthcare worker or teacher), presence of comorbidities (yes/no), and dominant SARS-CoV-2 variant circulating (Delta from 1 September 2021 to 28 December 2021, and then Omicron from 29 December 2021 to 10 January 2022). In the main analysis, we defined BIs as cases with any evidence of vaccination for more than 14 days prior to the date of their positive SARS-CoV-2 test result. In a sensitivity analysis, we considered only BIs who had completed their primary vaccination series (one dose for CanSino, Gamaleya, and Janssen; two doses for AstraZeneca, Moderna, Novavax, Pfizer, Sinopharma, and Sinovac).

To identify differences between variants, we compared Omicron BA.1 cases to Delta cases (reference group). For this analysis, the dependent variable was the variant and the independent variables remained the same as above (with the addition of vaccination status). Variants were presumed based on the date on which the sample was collected due to lack of sequencing to confirm the variant for all cases, where Delta cases were those prior to 28 December 2021, and Omicron cases were those after. This date corresponds to when Omicron BA.1 displaced Delta as the dominant variant (representing >50% of sequenced samples).

For both models, we included a random effect for the healthcare facility (File S1) where patients sought care and the SARS-CoV-2 test sample and demographic data were collected.

Statistical analysis was conducted in R (v. 4.3.0) with the packages tidyverse, reshape2, and ggplot2 (58–61).

### Genomic surveillance and epidemiology

#### SARS-CoV-2 diagnostics

Samples were analyzed by real-time RT-PCR to amplify a segment of the envelope gene or N gene and the constitutive ribonucleoprotein gene (62, 63). Samples with threshold cycles ≤32 were considered positive.

#### Methods for RNA extraction

Viral RNA was extracted by automated protocols using the MagNA Pure LC 2.0 instrument and the MagNA Pure LC Total Nucleic Acid Isolation Kit protocol (Roche).

#### SARS-CoV-2 sequencing

Samples were sequenced by the New York Genomic Center. SARS-CoV-2 targeted assay libraries were prepared using the Molecular Loop Viral RNA Target Capture Kit (Molecular Loop) in accordance with the manufacturer’s recommendations. Briefly, 6 µL of RNA was reverse transcribed and capture probes annealed in a 16 h incubation at 55°C. The probes were then enzymatically circularized to capture the viral genome and add Unique Molecular Indexes (UMI) followed by amplification of circularized cDNA targets using 27 cycles of PCR. Libraries were pooled by volume and cleaned up by magnetic bead purification. Final libraries were quantified using fluorescent-based assays, including PicoGreen (Life Technologies), Qubit Fluorometer (Invitrogen), and Fragment Analyzer (Advanced Analytics), and were sequenced on a NovaSeq 6000 sequencer with 2 × 150 bp reads.

#### SARS-CoV-2 genome assembly and lineage assignment

For each read pair, the two 5 bp UMIs located at the 5′ end of each mate were first clipped and combined into a single UMI tag. An additional 25 bp, corresponding to the molecular inversion probes, was also clipped from the 5′ end of each read. Next, read pairs that did not contain a single 19 bp seed k-mer in common with the SARS-CoV-2 genome reference (NC_045512.2) were discarded, and adapter sequences and low-quality bases (Q < 20) were trimmed from the 3′ end of the remaining reads, using Cutadapt (v2.10) (64). Processed read pairs were then merged using NGmerge (v0.2) (65), allowing for dovetailed alignments. The resulting single-end reads were mapped against the SARS-CoV-2 genome reference using BWA-MEM (v0.7.17) (66), and the resulting alignments were filtered using the following criteria: (i) reference span ≥50 bp, (ii) quality ≥60, and (iii) maximum soft-clip length on either end ≤30 bp. Next, reads representing the same original molecule were identified based on their shared UMI and alignment position and used to draw the molecule consensus sequence, considering base quality scores. Molecule sequences were then realigned to the SARS-CoV-2 genome reference using BWA-MEM (v0.7.17) (66). Finally, genome sequences were determined by molecule alignment pileup consensus calling with a minimum support of 5 molecules. We generated a total of 205 SARS-CoV-2 sequences from Yucatán, Mexico. SARS-CoV-2 lineages were assigned using Pangolin (v4.0.5) (https://github.com/cov-lineages/pangolin) (67).

### Data set compilation

To investigate the 205 study sequences in the global context, we obtained a representative background data set of the most genetically similar sequences using the AudacityInstant package of GISAID on 1 November 2022 (68).

We assigned the location of the sequences at the regional level, where those collected in Mexico and bordering countries (Belize, Guatemala, and the United States) were labeled by their country of collection, while all others were categorized by their continent of collection (Asia, Europe, North America, Oceania, and South America). Next, we applied a subsampling strategy using the Subsampling According to Metadata for Phylogenetic Inference (SAMPI) python tool (available at https://github.com/jlcherry/SAMPI) that selected a specific number of sequences per region per time, in order to build a data set that represented the overall circulating diversity. When sequences had the same country and collection date, we chose, in decreasing order of priority, those that were longer, had the month of collection rather than only the year, those with a smaller number of internal gaps with lengths not divisible by 3 (codons), and those with full dates of collection. A multiple sequence alignment of the combined study and background sequences was performed with Nextclade (69). A maximum-likelihood phylogenetic tree was inferred in IQ-TREE (v2.2.0_beta) (70) using the GTR+Gamma+I model and 1,000 ultrafast bootstrap replicates (71). The temporal signal was assessed in TempEst (v1.5.3) (72), and outlier sequences were removed. This resulted in a data set of 1,152 sequences (Africa, *n* = 68; Asia, *n* = 141; Belize, *n* = 1; Europe, *n* = 140; Guatemala, *n* = 27; Mexico, *n* = 140; Mexico study sequences, *n* = 205; North America, *n* = 140; Oceania, *n* = 62; South America, *n* = 140; the United States, *n* = 140; File S1) of which 403 sequences were of the Delta VOC and 749 were of Omicron VOC sequences.

#### Phylodynamic reconstruction

We constructed a temporally-aware phylogeny using the full-length genomes of 2,556 SARS-CoV-2 sequences using TreeTime (73), with a fixed molecular clock of 0.0008 substitutions/site/year and a standard deviation of 0.0004 (74). Tree tip dates were specified according to the sampling date, which provides a calibration method for sequence divergence. TreeTime utilizes the “dated tips” approach in which branch rates are auto-correlated along a constant population size as coalescent model (73). Scripts were called from the command line to perform standard tasks such as ancestral sequence inference, re-rooting of trees, time estimation, and ancestral reconstruction of the region trait using the “mugration” algorithm.

Methods and protocols for SARS-CoV-2 diagnostics were approved by the National Influenza Center, Instituto de Diagnóstico y Referencia Epidemiológicos (InDRE).

Methods and protocols for SARS-CoV-2 sequencing were approved by the New York Genomic Center in accordance with relevant guidelines and regulations.

## Data Availability

Data are provided within the article or supplemental information. Following is a direct link to sequencing data: https://epicov.org/epi3/epi_set/231031ax?main=true.

## SUPPLEMENTAL MATERIAL

Supplemental Material mbio.02432-25-s0001.pdf Inclusion and exclusion criteria; sensitivity analysis.

ASM does not own the copyrights to Supplemental Material that may be linked to, or accessed through, an article. The authors have granted ASM a non-exclusive, world-wide license to publish the Supplemental Material files. Please contact the corresponding author directly for reuse.
