# Erratum for Yuan et al., “Case-control and genomic epidemiology characterization of SARS-CoV-2 breakthrough infections during Delta-to-Omicron transition”

**DOI:** 10.1128/mbio.00455-26

**Published:** 2026-03-09

**Authors:** Erin Yuan, Chelsea L. Hansen, Sana Tamim, Samiah Kanwar, David J. Spiro, Refugio Gonzalez-Losa, Laura Conde-Ferraez, Pilar Granja-Pérez, Salha Villanueva-Jorge, Irma López-Martínez, Gisela Barrera-Badillo, André Corvelo, Samantha Fennessey, Michael C. Zody, Guadalupe Ayora-Talavera, Nidia S. Trovao

## ERRATUM

Volume 17, no. 2, e02432-25, 2026, https://doi.org/10.1128/mbio.02432-25. Byline: Samiah Kanwar’s name should appear as shown in this Erratum.

