# Supplementary material for: Case-control and genomic epidemiology characterization of SARS-CoV-2 breakthrough infections during the Delta-to-Omicron transition
Source: mBio. 2026 Jan 8;17(2):e02432-25. doi: 10.1128/mbio.02432-25 (PMC12892983; doi:10.1128/mbio.02432-25)
Supplement: Supplemental Material — Inclusion and exclusion criteria; sensitivity analysis. [file mbio.02432-25-s0001.pdf]

Supplementary Materials

Case-control and genomic epidemiology characterization of SARS-CoV-2 breakthrough infections during Delta to Omicron transition

Erin Yuan 1, Chelsea L. Hansen 2 3, Sana Tamim 4, Samiah Kanwar 5, David J. Spiro 2, Refugio Gonzalez-Losa 6, Laura Conde-Ferraez 6, Pilar Granja-Pérez 7, Salha Villanueva-Jorge 7, Irma López-Martínez 8, Gisela Barrera-Badillo 8, André Corvelo 9, Samantha Fennessey 9, Michael C. Zody 9, Guadalupe Ayora-Talavera 6*, Nidia S. Trovao 2*

Figure S1. Flowchart of inclusion and exclusion criteria and final sample size.

Figure S2. Odds ratio for sensitivity analysis of BIs with primary vaccination completed.

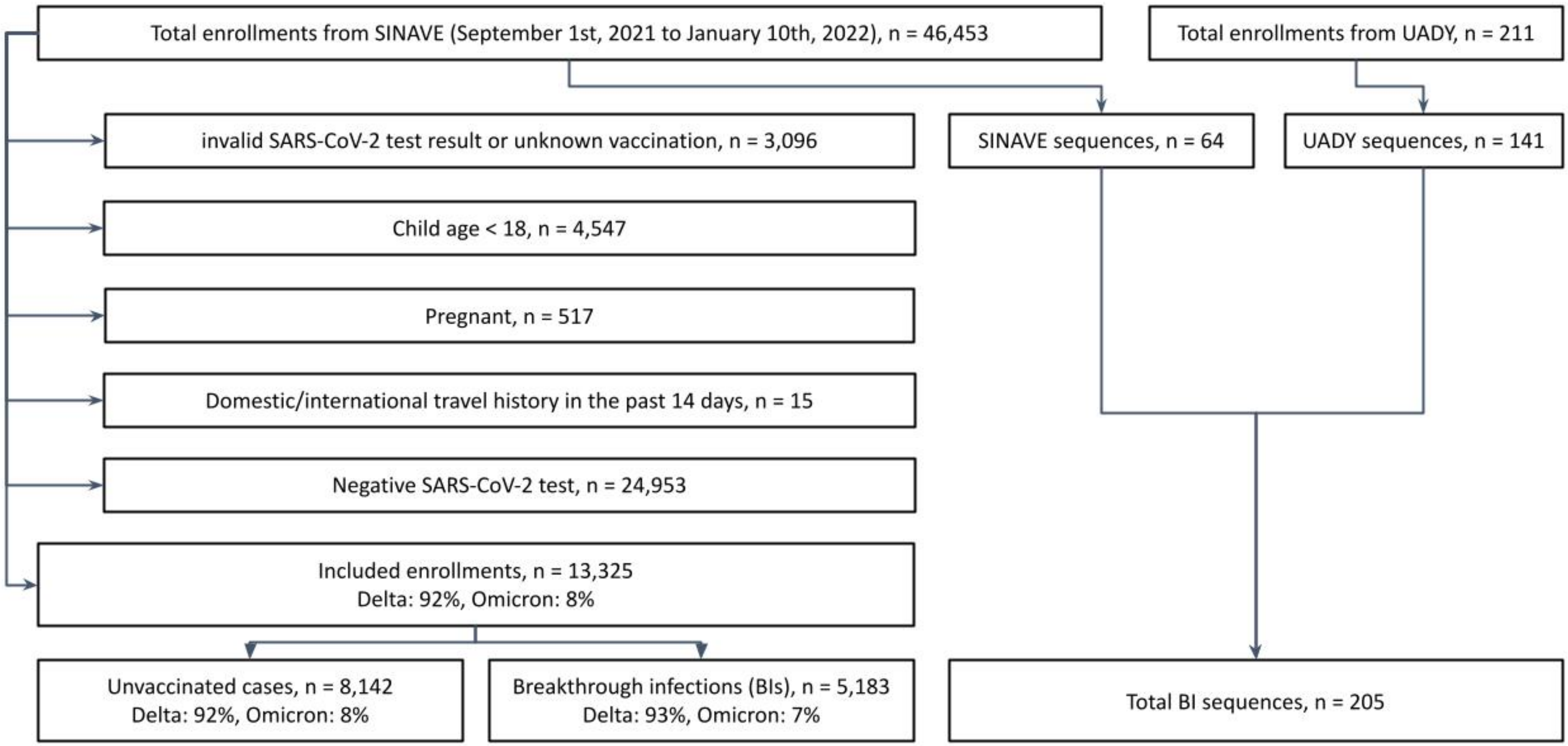
Total enrollments from SINAVE (September 1st, 2021 to January 10th, 2022), n = 46,453
Total enrollments from UADY, n = 211
invalid SARS-CoV-2 test result or unknown vaccination, n = 3,096
SINAVE sequences, n = 64
UADY sequences, n = 141
Child age < 18, n = 4,547
Pregnant, n = 517
Domestic/international travel history in the past 14 days, n = 15
Negative SARS-CoV-2 test, n = 24,953
Included enrollments, n = 13,325
Delta: 92%, Omicron: 8%
Unvaccinated cases, n = 8,142
Delta: 92%, Omicron: 8%
Breakthrough infections (BIs), n = 5,183
Delta: 93%, Omicron: 7%
Total BI sequences, n = 205

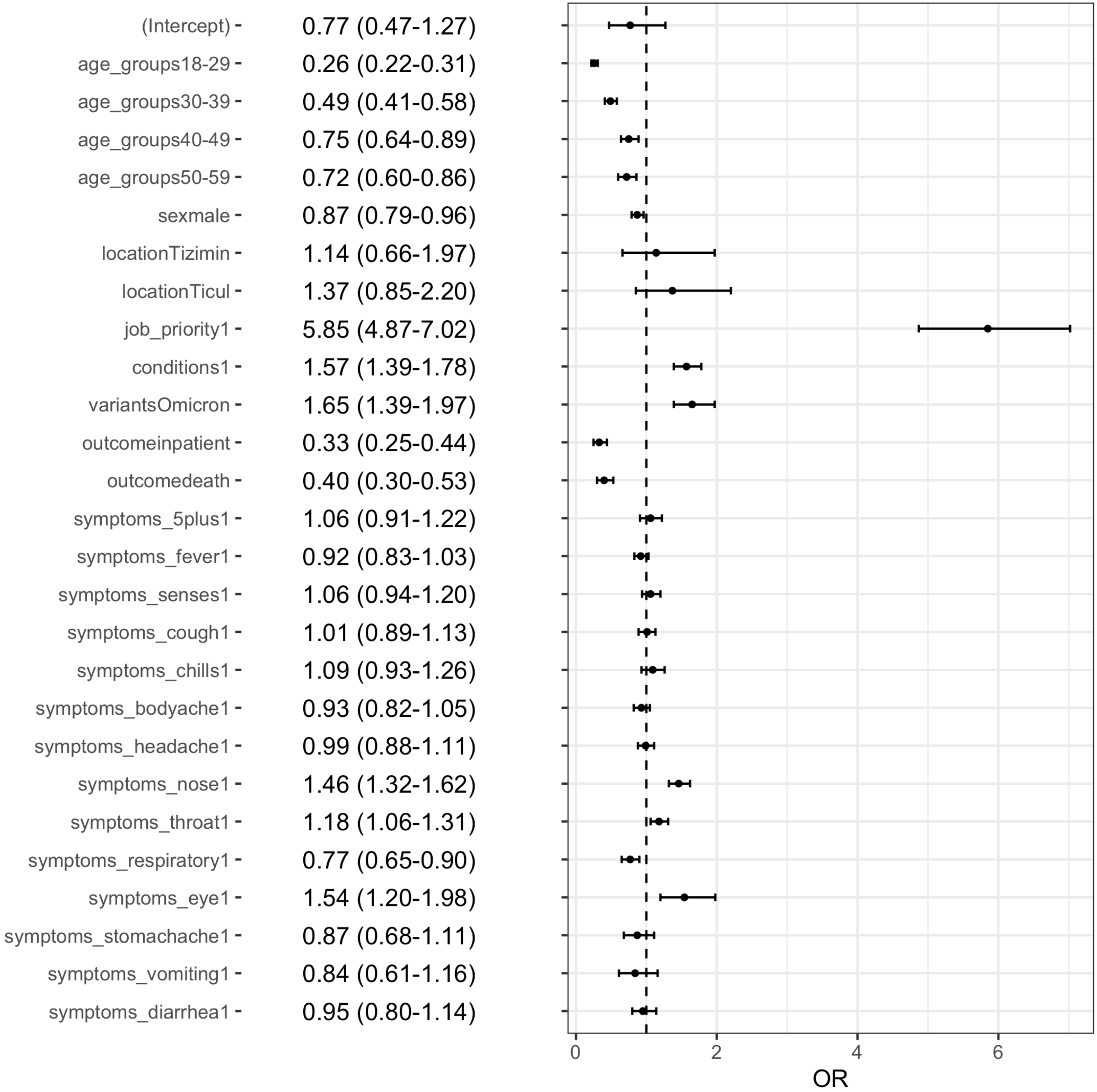
(Intercept) 0.77 (0.47-1.27)
age_groups18-29 0.26 (0.22-0.31)
age_groups30-39 0.49 (0.41-0.58)
age_groups40-49 0.75 (0.64-0.89)
age_groups50-59 0.72 (0.60-0.86)
sexmale 0.87 (0.79-0.96)
locationTizimin 1.14 (0.66-1.97)
locationTicul 1.37 (0.85-2.20)
job_priority1 5.85 (4.87-7.02)
conditions1 1.57 (1.39-1.78)
variantsOmicron 1.65 (1.39-1.97)
outcomeinpatient 0.33 (0.25-0.44)
outcomedeath 0.40 (0.30-0.53)
symptoms_5plus1 1.06 (0.91-1.22)
symptoms_fever1 0.92 (0.83-1.03)
symptoms_senses1 1.06 (0.94-1.20)
symptoms_cough1 1.01 (0.89-1.13)
symptoms_chills1 1.09 (0.93-1.26)
symptoms_bodyache1 0.93 (0.82-1.05)
symptoms_headache1 0.99 (0.88-1.11)
symptoms_nose1 1.46 (1.32-1.62)
symptoms_throat1 1.18 (1.06-1.31)
symptoms_respiratory1 0.77 (0.65-0.90)
symptoms_eye1 1.54 (1.20-1.98)
symptoms_stomachache1 0.87 (0.68-1.11)
symptoms_vomiting1 0.84 (0.61-1.16)
symptoms_diarrhea1 0.95 (0.80-1.14)
0
2
4
6
OR

SupFile 1. Patient questionnaire

SupFile 2. List of Healthcare facilities

SALUD Secretaría de Salud | IMSS | ISSSTE | PEMEX | SEDENA Secretaría de la Defensa Nacional | SEMAR Secretaría de Marina | SNDIF Sistema Nacional DIF | INPI Instituto Nacional de los Pueblos Indígenas

Estudio epidemiológico de caso sospechoso de enfermedad respiratoria viral

DATOS GENERALES

Nombre de la unidad:

Fecha de notificación en plataforma: dd/mm/aaaa Folio plataforma:

Apellido Paterno: Apellido Materno: Nombre (s):

Fecha de Nacimiento: Día: Mes: Año: CURP:

Sexo: Hombre: Mujer: ¿Está embarazada? Si No Meses de embarazo: Se encuentra en periodo de puerperio Si No Dias de puerperio

Nacionalidad: Mexicana: Extranjera: ¿Es migrante? Si No Pais de nacionalidad: País de origen:

Paises en tránsito en los últimos tres meses: 1 2 3 Otro: Fecha de ingreso a México:

País de nacimiento: Entidad federativa de nacimiento:

Entidad de Residencia: Municipio de residencia:

Localidad:

Calle: Número:

Entre qué calles: y

Colonia: C:P: Teléfono:

¿Se reconoce cómo índigena? Si No ¿Habla alguna lengua índigena? Si No

Ocupación:

¿Pertenece a alguna institución educativa?

DATOS CLÍNICOS

Servicio de ingreso: Tipo de paciente: 1=Ambulatorio 2=Hospitalizado

Fecha de ingreso a la unidad: dd/mm/aaaa Fecha de inicio de síntomas: dd/mm/aaaa

A partir de la fecha de inicio de síntomas:

¿Tiene o ha tenido alguno de los siguientes signos y síntomas?

	Si	No
Inicio súbito de los síntomas		
Fiebre		
Tos		
Cefalea		
Disnea		
Irritabilidad		
Dolor torácico		
Escalofrios		
Odinofagia		
Mialgias		
Artralgias		
Anosmia		
Disgeusia		
Rinorrea		
Conjuntivitis		

Co-morbilidad

	Si	No
Diabetes		
EPOC		
Asma		
Inmunosupresión		
Hipertensión		
VIH/SIDA		
Enfermedad cardiovascular		
Obesidad		
Insuficiencia renal crónica		
Tabaquismo		
Otros		

Espeficique otros:

Otros síntomas	Si	No
Ataque al estado general		
Diarrea		
Polipnea		
Dolor Abdominal		
Vómito		
Cianosis		

Diagnóstico probable: 1=Enfermedad tipo influenza (ETI)
2=Infección respiratoria aguda grave (IRAG)

*ETI es considerada como Enfermedad respiratoria leve

TRATAMIENTO

¿Desde el inicio de los síntomas ha recibido tratamiento con antipiréticos? Si [ ] No [ ]

¿Desde el inicio de los síntomas ha recibido tratamiento con antivirales? Si [ ] No [ ]

Si la respuesta fue afirmativa:

Seleccione el antiviral: [ ] 1=Amantadina 2=Rimantadina 3=Oseltamivir 4=Zanamivir 5=Otro, Especifique otro: [ ]

¿Cuándo se inicio el tratamiento antiviral? [ ] dd/mm/aaaa

En la unidad médica:

¿Se inicia tratamiento con antimicrobianos ? Si [ ] No [ ]

¿Se inicia tratamiento con antivirales? Si [ ] No [ ]

Seleccione el antiviral: [ ] 1=Amantadina 2=Rimantadina 3=Oseltamivir 4=Zanamivir 5=Otro, Especifique otro: [ ]

ANTECEDENTES EPIDEMIOLÓGICOS

¿Tuvo contacto con casos con enfermedad respiratoria en las ultimas dos semanas? Si [ ] No [ ]

Durante las semanas previas al inicio de los síntomas tuvo contacto con:

	Si	No
Aves		
Cerdos		

Otro animal [ ]

¿Realizó algún viaje 7 días antes del inicio de signos y síntomas? Si [ ] No [ ]

País: [ ] Ciudad: [ ]

¿Recibió la vacuna contra influenza en último año? Si [ ] No [ ] Fecha de vacunación: [ ] dd/mm/aaaa

¿Recibió la vacuna contra COVID-19 en último año? Si [ ] No [ ] Marca de la vacuna: [ ]

*Coloque el numero de acuerdo a la marca de la vacuna que indique el caso

¿Cuantas dosis recibió? 1 [ ] 2 [ ]

** Al marcar dos dosis, tendrá que colocar las dos fechas de vacunación de forma obligatoria*

1era dosis Fecha de vacunación: [ ] dd/mm/aaaa

2da dosis Fecha de vacunación: [ ] dd/mm/aaaa

1	Pfizer BioNTech	6	Janssen (Johnson&Johnson)	11	Convidencia
2	AstraZeneca	7	Sinopharma		
3	CanSino	8	Novavax		
4	Moderna	9	No recuerda		
5	Gamaleya "Sputnik V"	10	Sinovac		

MUESTRA PARA ANTÍGENO DE COVID-19

¿Se le tomó muestra al paciente? Si [ ] No [ ] Fecha de toma de muestra: [ ] dd/mm/aaaa

Resultado de la muestra de antígeno: [ ]

MUESTRA DE LABORATORIO PARA PCR

¿Se le tomó muestra al paciente? Si [ ] No [ ]

Laboratorio al que se solicita el procesamiento de la muestra: [ ]

Tipo de muestra: [ ] 1=Exudado faríngeo 2=Exudado Nasofaríngeo 3=Lavado bronquial 4=Biopsia de pulmón

Fecha de toma de muestra: [ ] dd/mm/aaaa

Resultado: [ ]

EVOLUCIÓN

Evolución: [ ] 1=Alta 2=En tratamiento/Referencia/Seguimiento domicilario/Seguimiento terminado 3=Caso grave 4=Caso no grave 5=Defunción*

Si el caso se da de alta: Especifíque la evolución: [ ] 1=Mejoría 2=Curación 3=Voluntaria 4=Traslado

¿El caso está o estuvo ingresado en la UCI durante la enfermedad? Si [ ] No [ ]

¿El caso está o estuvo intubado en algún momento durante la enfermedad? Si [ ] No [ ]

¿El caso tiene o tuvo diagnóstico de neumonia durante la enfermedad? Si [ ] No [ ]

Fecha de egreso: [ ] dd/mm/aaaa

Se trató de un caso positivo a COVID-19 por asociación o dictaminación clínica-epidemiológica:*

** Marque solo una de las siguientes opciones de respuesta con una X*

a. Confirmado a COVID-19 por asociación clínica-epidemiológica [ ]
b. Confirmado a COVID-19 por dictaminación clínica-epidemiológica (aplica solo en defunciones) [ ]
c. No (es decir ninguna de las anteriores) [ ]

Defunción: Fecha de defunción: [ ] dd/mm/aaaa

Folio de certificado de defunción [ ] *Defunción por influenza o COVID-19 Si [ ] No [ ]

*Anexar copia de certificado de defunción si cumple con definición operacional de caso sospechoso de enfermedad respiratoria viral

Nombre y cargo de quien elaboró ____________________

Nombre y cargo de quien autorizó ____________________

Fecha de elaboración: [ ] dd/mm/aaaa

Supplementary File 2

1. C.S. PETO - SSA
2. C.S. TICUL - SSA
3. CENTRO DE ESPECIALIDADES MEDICAS
4. CENTRO DE SALUD CHICHIMILA - SSA
5. CENTRO DE SALUD RIO LAGARTOS - SSA
6. CENTRO DE SALUD BUCTZOTZ - SSA
7. CENTRO DE SALUD CHIKINDZONOT - SSA
8. CENTRO DE SALUD CON SERVICIOS AMPLIADOS DE PROGRESO - SSA
9. CENTRO DE SALUD DE CENOTILLO - SSA
10. CENTRO DE SALUD DE CHELEM PUERTO - SSA
11. CENTRO DE SALUD DE CHEMAX - SSA
12. CENTRO DE SALUD DE KOMCHEN - SSA
13. CENTRO DE SALUD DE PANABA - SSA
14. CENTRO DE SALUD DE TEKOM - SSA
15. CENTRO DE SALUD DZITAS - SSA
16. CENTRO DE SALUD KANASIN - SSA
17. CENTRO DE SALUD KIMBILA - SSA
18. CENTRO DE SALUD MUXUPIB - SSA
19. CENTRO DE SALUD PISTE - SSA
20. CENTRO DE SALUD SANTA ROSA - SSA
21. CENTRO DE SALUD SUCILA - SSA
22. CENTRO DE SALUD TEKAX - SSA
23. CENTRO DE SALUD TEMOZON - SSA
24. CENTRO DE SALUD TIZIMIN - SSA
25. CENTRO DE SALUD UAYMA - SSA
26. CENTRO DE SALUD VALLADOLID - SSA
27. CENTRO DE SALUD YAXCABA - SSA
28. CENTRO SALUD MERIDA - SSA
29. CLINICA DE MERIDA - PRIVATE
30. CLINICA HOSPITAL MERIDA -ISSSTE
31. GRUPO MEDICO DE MERIDA SA DE CV - PRIVATE
32. HGR 12 BENITO JUAREZ - IMSS
33. HGSMF 3 MOTUL - IMSS
34. HGSMF 46 UMAN - IMSS
35. HGSMF 5 TIZIMIN - IMSS
36. HOSPITAL DE LA AMISTAD (COREA-MEXICO)- PRIVATE
37. HOSPITAL DE LA COMUNIDAD EN PETO - SSA
38. HOSPITAL FARO DEL MAYAB S.A.P.I. DE C.V. - PRIVATE
39. HOSPITAL GENERAL AGUSTIN O´HORAN - SSA
40. HOSPITAL GENERAL DE VALLADOLID - SSA
41. HOSPITAL GENERAL EN TEKAX - SSA
42. HOSPITAL GENERAL REGIONAL NUMERO 1 - IMSS

43. HOSPITAL GENERAL SAN CARLOS - SSA
44. HOSPITAL MILITAR REGIONAL DE MERIDA - SEDENA
45. HOSPITAL REGIONAL DE ALTA ESPECIALIDAD DE LA PENINSULA DE YUCATAN - SSA
46. HR ACANCEH - IMSS
47. HR IZAMAL - IMSS
48. HR MAXCANU - IMSS
49. HR OXKUTZCAB - IMSS
50. INSTITUTO MEDICO PANAMERICANO / CMA - PRIVATE
51. MERIDA - ISSSTE
52. SOCIEDAD MEDICA GARCIA GINERES - PRIVATE
53. STAR MEDICA - PRIVATE
54. UMAA 1 MERIDA (AUT) -IMSS
55. UMAE NUMERO 25 - IMSS
56. UMF 13 CHUBURNA - IMSS
57. UMF 14 KANASIN - IMSS
58. UMF 17 MAXCANU - IMSS
59. UMF 41 ACANCEH - IMSS
60. UMF 6 COL. YUCATAN - IMSS
61. UMF 8 TZUCACAB - IMSS
62. UMF 16 KOMCHEN - IMSS
63. UMF 20 CAUCEL - IMSS
64. UMF 21 SAMAHIL - IMSS
65. UMF 31 IZAMAL - IMSS
66. UMF 49 TIXKOKOB - IMSS
67. UMF 52 MERIDA - IMSS
68. UMF 54 TICUL - IMSS
69. UMF 55 TEKAX - IMSS
70. UMF 56 MERIDA - IMSS
71. UMF 57 LA CEIBA - IMSS
72. UMF 58 MERIDA - IMSS
73. UMF 59 MERIDA - IMSS
74. UMF 60 PONIENTE - IMSS
75. UMF 7 LAS COLORADAS - IMSS
76. UMFH 19 HUNUCMA - IMSS
77. UMFH 2 PROGRESO - IMSS
78. UMFH 4 VALLADOLID - IMSS
79. UMFH 50 CONKAL - IMSS

Supplementary Table 1. Source of SARS-CoV-2 VOCs leading to breakthrough infections in Yucatán, Mexico. Normalized transmission rates (as percentage) across regions colored from lowest (green) to highest (red) contribution in percentage to breakthrough infections in Yucatán.

	From									
	Africa	Asia	Belize	Europe	Guatemala	Mexico	North America	Oceania	South America	USA
All	5.77	8.43	1.48	5.42	8.75	23.16	16.09	10.76	11.25	8.89
Delta	2.54	3.82	2.53	7.17	14.83	27.93	13.19	2.53	12.53	12.91
Omicron	7.80	11.49	0.00	5.36	1.79	22.04	19.86	10.33	14.76	6.57

SUPPLEMENTAL TABLE

Data Availability

GISAID Identifier:	EPI_SET_231031ax
doi:	10.55876/gis8.231031ax

All genome sequences and associated metadata in this dataset are published in GISAID's EpiCoV database. To view the contributors of each individual sequence with details such as accession number, Virus name, Collection date, Originating Lab and Submitting Lab and the list of Authors, visit 10.55876/gis8.231031ax

Data Snapshot

- EPI_SET_231031ax is composed of 1,201 individual genome sequences.
- The collection dates range from 2021-01-03 to 2022-06-11;
- Data were collected in 80 countries and territories;
- All sequences in this dataset are compared relative to hCoV-19/Wuhan/WIV04/2019 (WIV04), the official reference sequence employed by GISAID (EPI_ISL_402124). Learn more at https://gisaid.org/WIV04.
